# Joint effects of physical activity and sleep quality on all-cause and cardiovascular disease mortality in stroke survivors: a population-based cohort study from the UK-Biobank

**DOI:** 10.1186/s12889-025-22588-5

**Published:** 2025-04-23

**Authors:** Yanhan Zhu, Bo Chen, Minghui Qin, Jing Yang, Mei Hu, Jingjing Zeng, Menglin Fan, Ke Wang, Liying Chang, Shaoyong Xu

**Affiliations:** 1 Department of Neurology, Xiangyang Central Hospital, Affiliated Hospital of Hubei University of Arts and Science, Xiangyang, Hubei China; 2 Center for Clinical Evidence-Based and Translational Medicine, Xiangyang Central Hospital, Affiliated Hospital of Hubei University of Arts and Science, Xiangyang, Hubei China; 3 Department of Traditional Chinese Medicine, Xiangyang Central Hospital, Affiliated Hospital of Hubei University of Art and Science, Xiangyang, Hubei China; 4 Department of Preventive Medicine, Medical College, Hubei University of Arts and Science, Xiangyang, Hubei China; 5 Department of Endocrinology, Xiangyang Central Hospital, Affiliated Hospital of Hubei University of Arts and Science, Xiangyang, Hubei China

**Keywords:** Physical activity, Sleep, Mortality, Stroke survivors

## Abstract

**Background:**

Stroke survivors exhibit a higher prevalence of sleep disturbances and physical activity (PA) deficiencies. The joint effects of the two behaviors on mortality risk among stroke survivors remains unclear. This study aimed to explore the joint association of PA and sleep quality with the all-cause and cardiovascular disease ( CVD ) mortality risk in stroke survivors.

**Methods:**

A total of 5,507 stroke survivors from the UK Biobank were included to assess the independent or joint associations of sleep score and PA with mortality. PA levels were categorized as meeting recommended moderate-to-vigorous physical activity (MVPA) and not meeting recommended MVPA. Sleep quality was classified as healthy, poor/intermediate based on a novel sleep score, leading to the identification of four distinct PA-sleep combinations. Cox proportional hazard models were employed to estimate hazard ratio (HR) for all-cause and cardiovascular disease (CVD) mortality, with data ascertained through October 2021. The dose-response relationship between PA or sleep duration and mortality risk were explored by plotting restricted cubic splines. Sensitivity analyses were conducted to examine the robustness of the results.

**Results:**

After an average follow-up of 12.55 years, healthy sleep score group were associated with an decreased all-cause mortality risk compared to poor/intermediate sleep score group (HR: 0.873; 95% CI: 0.767–0.995). Compared to individuals who did not meet the recommended MVPA levels, those who did achieve the recommended MVPA levels was exhibited a significantly lower risk of all-cause mortality (HR, 0.729; 95% CI, 0.640–0.831) and CVD mortality (HR, 0.786; 95% CI, 0.627–0.986). PA levels exhibit an L-shaped association with mortality(cut off value = 2,000 MET-minutes per week). Participants meeting MVPA recommendations and/or reporting healthy sleep scores reduced 28.5–35.9% risk of all-cause mortality.

**Conclusions:**

Poor sleep quality is associated with a elevated risk of all-cause mortality. Stroke survivors meeting recommended MVPA levels exhibit lower mortality risk, even among those with poor sleep quality. Future intervention studies are needed to establish whether increasing PA to recommended levels among stroke survivors directly reduces mortality risk linked to poor sleep quality.

**Supplementary Information:**

The online version contains supplementary material available at 10.1186/s12889-025-22588-5.

## Introduction

Stroke is the second leading cause of death worldwide according to a Global Burden of Disease study. Furthermore, the risk of death from cardiovascular disease (CVD) and other diseases is particularly increased among stroke patients [1]. Stroke survivors have a higher incidence of sleep disturbance and physical activity (PA) deficiency than the general population due to the chronic physical and psychological effects of their disabilities [2–3]. In particular, the association between sleep and PA as potential factors leading to adverse outcomes in stroke patients is complex. Therefore, it is important to assess the relationship between sleep, PA levels, and their combined effects on disease outcome and mortality in stroke survivors.

It is well documented that post-stroke sleep disturbance is associated with increased disease severity and risk of death after stroke [4–6]. However, studies on sleep and risk of death in stroke survivors have focused on individual sleep factors (e.g., sleep duration [6], insomnia [7], snoring [8]). The lack of standardized sleep measures may hinder investigations of sleep in relation to health and its joint effects with other behaviors. Considering various sleep factors together may be more easily interpreted and translated into recommendations for secondary prevention in stroke survivors than individual sleep parameters. Recently, Fan et al. developed a novel scoring system that integrated sleep duration and sleep quality into a single sleep measure and demonstrated its ability to identify high-risk populations [9]. However, this score is currently used mainly in the general population [4], and to our knowledge, there are no studies involving stroke survivors.

In addition, it is well documented that high PA levels in pre-stroke patients are associated with reduced disease severity and all-cause mortality risk after stroke [10]. For post-stroke survivors, there is evidence that appropriate individualized PA programs after stroke is beneficial for functional recovery and health status [3, 11–13]. However, there is a paucity of studies exploring the relationship between the maintenance of adequate PA levels in stroke survivors and the risk of death. Only one study has suggested that achieving adequate PA levels may reduce all-cause mortality, stroke recurrence, and other adverse events in ischemic stroke survivors, but these data are limited by a large study bias, resulting in a potential association that remains unclear [14]. Moreover, previous study has been shown that PA levels and sleep may be interdependent and affect health status through improving metabolic adaptations and maintaining stable circadian rhythms [15–17]. However, the potential joint effects of these two key behaviors in stroke survivors remain largely unknown. Therefore, in addition to investigating the independent health effects of PA and sleep in stroke survivors, we also investigated the joint association of PA and novel composite sleep scores with the risk of all-cause and CVD mortality in stroke survivors.

## Methods

### Population

The UKB is a very large and detailed prospective cohort with 502,490 participants aged 40–69 years recruited between 2006 and 2010. All participants completed a touch-screen questionnaire, had physical measurements taken, and provided blood, urine, and saliva samples at baseline at one of 22 assessment centers throughout England, Scotland, and Wales (https://www.ukbiobank.ac.uk/). The UKB received ethics approval from the North West Multi-Center Research Ethics Committee (Ref 11/NW/03820). All participants provided written informed consent for the study. The present study was performed under application number 92,014.

Stroke diagnosis was derived from self-reported physician diagnosis. There were 9,260 participants with self-reported stroke at the baseline survey. Firstly, 1,780 participants with severe heart disease or cancer at baseline were excluded, Secondly, 205 participants who died during baseline recruitment were excluded. Additionally, we excluded those with missing sleep score data (*n* = 10) and missing PA data (*n* = 1,768), leaving a final sample of 5,507 participants for investigating the independent and combined association of PA and sleep scores with the risk of all-cause and CVD mortality in stroke survivors (Figure S1).

### Outcomes

The primary endpoint was all-cause mortality with a secondary endpoint of CVD mortality. Using the International Classification of Disease 10th version (ICD, 10th), mortality due to CVD was defined as codes I00–I99 [7], while all-cause mortality was identified as mortality from any cause. Date of death was obtained from death certificates through data linkage with national datasets from the National Health Service (NHS) Information Centre (England and Wales) and the NHS Central Register Scotland (Scotland). Data on mortality were accessible until October 2021. As a result, mortality follow-up was suspended on this date or the date recorded for death.

### Exposures

PA was quantified using the modified short-form International Physical Activity Questionnaire, which assesses the duration and frequency of PA in leisure time. We divided PA into two groups: moderate to vigorous physical activity (MVPA) recommended versus not recommended according to whether MVPA levels met World Health Organization (WHO) standard recommendations (≥ 150 min of moderate PA, or ≥ 75 min of vigorous PA, or equivalent combinations of both throughout the week) [18]. Weekly PA was summarised using weekly total MET, calculated by multiplying the MET value of activity by the number of PA hours per week. Based on the lower and upper limits of the WHO PA guideline, PA was categorised as low (0 to < 600 MET-mins/week), medium (600 to < 1200 MET-mins/week) and high (≥ 1200 MET-mins/week).

We used a novel sleep score that included five sleep factors: chronotype (the propensity to sleep at a particular time during a 24-hour period), duration, insomnia, snoring, and excessive daytime sleepiness [12]. Early chronotype, sleep 7–8 h per day, absent or rare insomnia symptoms, no snoring, and no frequent daytime sleepiness were considered low-risk sleep characteristics. Each sleep factor was assigned a score of 1 if present and 0 if absent. The final sleep scores ranged from 0 to 5, with higher scores indicating a healthier sleep pattern. Sleep scores were then classified into three groups: “healthy sleep” (sleep score ≥ 4); “intermediate sleep” (sleep score 2–3); and “poor sleep”(sleep score ≤ 1) (Table S1).

Ultimately, given the small sample size of individuals with poor sleep quality, this study categorized both poor and intermediate sleepers into a single group for the analysis. The PA and sleep-score categories were combined to investigate their combined associations using the following categories: (I) Poor/Intermediate sleep scores group and not meeting the recommended MVPA group; (II) Poor/Intermediate sleep scores group and meeting the recommended MVPA group; (III) Healthy sleep score group and not meeting the recommended MVPA group; and (IV) Healthy sleep score group and meeting the recommended MVPA group.

### Covariates

To reduce the effect of potential confounding, demographics and contextual covariates were selected based on previous literature [7], and included age, sex, body mass index (BMI), socioeconomic status, mental health issue, employment, smoking status, alcohol consumption, sedentary behaviour, living alone, hypertension, diabetes, and PA or sleep scores when applicable. Table S2 describes the definitions in detail.

### Statistics and data analysis

To avoid bias that may be caused by missing variables, we estimated missing values through multiple imputations based on chained equations. Descriptive statistics were presented stratified by sleep score and PA level, and continuous variables were expressed as mean ± standard deviation; categorical variables are expressed as n (%). Categorical data were analyzed using the χ^2^ test or Fisher exact-probability test. Student’s *t* test, analysis of variance (ANOVA), or the Wilcoxon rank-sum test was used to analyze the differences between groups for continuous and one-way ordered data.

The proportional hazards assumption was validated using Schoenfeld residuals. Cox proportional hazards regression models were used to estimate the independent and joint associations between PA and sleep scores with mortality. The time indicator was the follow-up time from baseline (2006–2010) to death or cut-off date (October 31, 2021). When exploring independent effects, not meeting the recommended MVPA group and poor/intermediate sleep scores group were used as references, respectively; when exploring the joint effect of both, the combination of poor/intermediate sleep scores group and not meeting the recommended MVPA group was used as reference. Multiple covariates were selected and adjusted for in both models. In model 1, we adjusted for age and sex; in model 2, we further adjusted for BMI, mental health issues, employment status, smoking status, alcohol consumption status, sedentary behavior, living alone, socioeconomic status, and vascular risk factors (hypertensive disease, diabetes), and sleep scores or physical activity levels as appropriate. We explored the dose-response relationship between PA or sleep duration and mortality risk by plotting restricted cubic splines (RCS) (konts = 4) using PA or sleep duration as continuous variables. Additionally, when examining independent effects, we also further investigated the relationship between individual sleep patterns (chronotype, duration, insomnia, snoring, and excessive daytime sleepiness) or the physical activity levels and mortality. To explore the stability of the results, we conducted the following sensitivity analyses: (1) We examined potential interaction effects by performing additive or multiplicative interactions analyses between the two primary exposures (PA and sleep scores); (2) We also excluded stroke patients prior to the baseline survey based on the date of their first reported stroke, utilizing the UK Biobank codes (UKB codes: 42006); (3) Given potential competing risks among study outcomes, we further validated the association between the two primary exposures (MVPA and sleep scores) and cardiovascular mortality in a competing risk model. All statistical tests were two-sided and performed using SAS 9.4 (SAS Institute, Cary, NC) and R-4.4.1 (http://www.R-project.org/); *P* < 0.05 was considered to be statistically significant.

## Results

### Baseline characteristics

In this study, data were largely complete, with only 1.8% of sedentary time data and 1.5% of BMI data missing. The missing data for all other covariates were below 1% (Figure S2). The median follow-up period for the joint association between sleep score and PA with mortality in this study was 12.55 years (interquartile range, 11.65–13.36 years; with a follow-up of 66,275.91 person-years). Table 1 and Table S3 show the baseline characteristics of the study participants with different sleep scores and MVPA categories, respectively. The 2,537 (46.06%) of participants were classified as healthy sleep score group. Compared with the poor/intermediate-sleep score group, participants with healthy sleep score had consistently lower BMI, less sedentary time, lower rates of mental health problems. and lower PA levels. More than half of the participants with healthy sleep score (1,331, 52.46%) achieved the recommended MVPA. Conversely, the group not meeting the MVPA recommendations had a higher BMI; had a longer sedentary time; and had higher rates of mental health problems, current smoking, and comorbid hypertension or diabetes.

**Table 1:** Baseline characteristics of the study participants by categories of sleep score

Characteristics	Sleep scores ^a^			
	All (N = 5, 507)	Poor/Intermediate (N = 2, 970)	Healthy (N = 2, 537)	*p*
age, mean (SD)	60.12 (7.06)	60.06 (6.90)	60.19 (7.25)	0.499
Sex, n (%)				0.049
Male	3,304 (60.00)	1,818 (61.21)	1,486 (58.57)	
Female	2,203 (40.00)	1,152 (38.79)	1,051 (41.43)	
Body Mass Index (kg/m^2^), mean (SD)	28.52 (5.10)	29.03 (5.35)	27.92 (4.74)	< 0.001
Body Mass Index (kg/m^2^), n (%)				<0.001
Underweight (< 18.5 kg/m2)	102 (1.85)	60 (2.02)	42 (1.66)	
Normal weight (18.5–24.9 kg/m2)	1,263 (22.93)	583 (19.63)	678 (26.72)	
Overweight (25.0–29.9 kg/m2)	2,343 (42.55)	1,250 (42.09)	1,094 (43.12)	
Obese (≥ 30.0 kg/m2)	1,799 (32.67)	1,077 (36.26)	723 (28.50)	
Socioeconomic status, mean (SD)	-0.74 (3.33)	-0.50 (3.43)	-1.02 (3.20)	<0.001
Sedentary behaviour (hour/day), mean (SD)	5.16 (2.73)	5.47 (2.86)	4.80 (2.51)	<0.001
Mental health issue, n (%)				<0.001
No	3,284 (59.63)	1,631 (54.92)	1,653 (65.16)	
Yes	2,223 (40.37)	1,339 (45.08)	884 (34.84)	
Employment status, n (%)				<0.001
Retired/not in the workforce	3,876 (70.38)	2,153 (72.49)	1,723 (67.91)	
Employed not in shift work	1,345 (24.42)	650 (21.89)	695 (27.39)	
Employed in night shift work	135 (2.45)	76 (2.56)	59 (2.33)	
Employed in day shift work	151 (2.74)	91 (3.06)	60 (2.36)	
Cigarette smoking, n (%)				<0.001
Never	2,366 (42.96)	1,153 (38.82)	1,213 (47.81)	
Previous smoker	2,324 (42.20)	1,294 (43.57)	1,030 (40.60)	
Current smoker	817 (14.84)	523 (17.61)	294 (11.59)	
Alcohol consumption, n (%)				0.009
Never	313 (5.68)	159 (5.35)	154 (6.07)	
Previous drinker	380 (6.90)	233 (7.85)	148 (5.83)	
Current drinker	4,814 (87.42)	2,578 (86.80)	2,235 (88.10)	
Living alone, n (%)				0.019
No	4,193 (76.14)	2,224 (74.88)	1,969 (77.61)	
Yes	1,314 (23.86)	746 (25.12)	568 (22.39)	
Hypertension history, n (%)				0.004
No	2,917 (52.97)	1,519 (51.14)	1,398 (55.10)	
Yes	2,590 (47.03)	1,451 (48.86)	1,139 (44.90)	
Diabetes history, n (%)				<0.001
No	4,821 (87.54)	2,534 (85.32)	2,287 (90.15)	
Yes	686 (12.46)	436 (14.68)	250 (9.85)	
Physical activity ^b^				<0.001
low (0 < 600 MET-min/week)	1,374 (24.95)	860 (28.96)	514 (20.26)	
medium (600 < 1200 MET-min/week)	974 (17.69)	521 (17.54)	453 (17.86)	
High (≥ 1200 MET-min/week)	3,159 (57.36)	1,589 (53.50)	1,570 (61.88)	
MVPA (min/week) (mean ± SD)				<0.001
Not recommended MVPA, n (%)	2,849 (51.73)	1,643 (55.32)	1,206 (47.54)	
Recommended MVPA, n (%)	2,658 (48.27)	1,327 (44.68)	1,331 (52.46)	

Note: Continuous variables are presented as mean ± SD; categorical variables are presented as n (%). ^a^ Sleep scores were categorised into: poor/intermediate, 0 ~ 3; healthy, 4 ~ 5. ^b^ Physical active were categorization based on public health guidelines: low (< 600 MET-mins/week); medium (600 to < 1200 MET-mins/week); and high (≥ 1200 MET-mins/week). Recommended MVPA (≥ 150 min of MPA, or ≥ 75 min of VPA, or equivalent combinations of both throughout the week). SD, standard deviation; n, total number; MVPA, moderate-to-vigorous physical activity; MPA, Moderate- intensity physical activity; VPA.Vigorous-intensity physical activity

### Independent associations of PA and sleep scores with mortality

Table 2, Table S4, and Table S5 show the independent association of sleep scores and PA with mortality risks. A total of 970 (17.61%) participants died, of whom 317 (32.68%) died of CVD. Independent associations of sleep scores and PA with mortality are shown in Table 2. In the full model, the healthy sleep score group was associated with an decreased risk of all-cause mortality compared to individuals with the poor/intermediate sleep score group (hazard ratio (HR), 0.873; 95% CI, 0.767–0.995, *p-trend* < 0.05). However, sleep score was not significantly associated with the risk of CVD mortality after full adjustment. In examining the relationship between individual sleep patterns and mortality, we found that prolonged sleep duration was significantly associated with an increased risk of all-cause mortality (hazard ratio [HR], 1.325; 95% confidence interval [CI], 1.118–1.572). The RCS analysis reveals that an increase in sleep duration beyond 8 h is associated with a significant elevation in the risk of all-cause mortality and CVD mortality (Figure S3). In addition, more frequent daytime dozing was associated with a higher risk of all-cause mortality in individual sleep patterns (HR, 1.192; 95% CI, 1.005–1.531). This was likewise observed for CVD mortality outcomes (Table S4).

**Table 2:** The independent (and mutually adjusted) associations of physical activity and sleep scores with mortality ^a^

	Number of cases	Number of total	Person-years	Incidence density (1000 person-year)	Model 1	Model 2
All-cause Mortality						
Sleep Scores (categorical)						
Poor/Intermediate	571	2,970	35,582.15	16.04	Ref.	Ref.
Healthy	399	2,537	30,693.77	12.99	0.791 (0.696, 0.899)^*^	0.873 (0.767, 0.995)^*^
Sleep Scores (continuous)					0.896 (0.846, 0.949)^*^	0.949 (0.895, 0.999)^*^
Physical Activity (categorical)						
Not recommended MVPA	576	2,849	34,090.27	16.90	Ref.	Ref.
Recommended MVPA	394	2,658	32,185.64	12.24	0.683 (0.601, 0.776)^*^	0.729 (0.640, 0.831)^*^
Physical Activity (continuous)					0.998 (0.998, 0.999)^*^	0.999 (0.999, 1.000)^*^
**CVD Mortality**						
Sleep Scores (categorical)						
Poor/Intermediate	175	2,970	35,582.15	4.92	Ref.	Ref.
Healthy	142	2,537	30,693.77	4.62	0.916 (0.734, 1.143)	0.998 (0.802, 1.260)
Sleep Scores (continuous)					0.929 (0.840, 1.028)	0.985 (0.888, 1.095)
Physical Activity (categorical)						
Not recommended MVPA	181	2,849	34,090.27	5.31	Ref.	Ref.
Recommended MVPA	136	2,658	32,185.64	4.22	0.747 (0.598, 0.934)^*^	0.786 (0.627, 0.986)^*^
Physical Activity (continuous)					0.998 (0.998, 0.999)^*^	0.999 (0.999, 1.000)^*^

^a^ Physical activity were categorization based on public health guidelines: Recommended MVPA (≥ 150 min of MPA, or ≥ 75 min of VPA, or equivalent combinations of both throughout the week); Not recommended MVPA. Sleep scores were categorized into: poor/intermediate, 0 ~ 3; healthy, 4 ~ 5. Model1: Adjusted for age and sex; Model2: Further adjusted for BMI, mental health issue, employment, smoking status, drinking, sedentary behaviour, alone, hypertension, diabetes and mutually adjusted for sleep scores or physical activity levels as appropriate. ^*^: *p* < 0.05

**Fig. 1:**
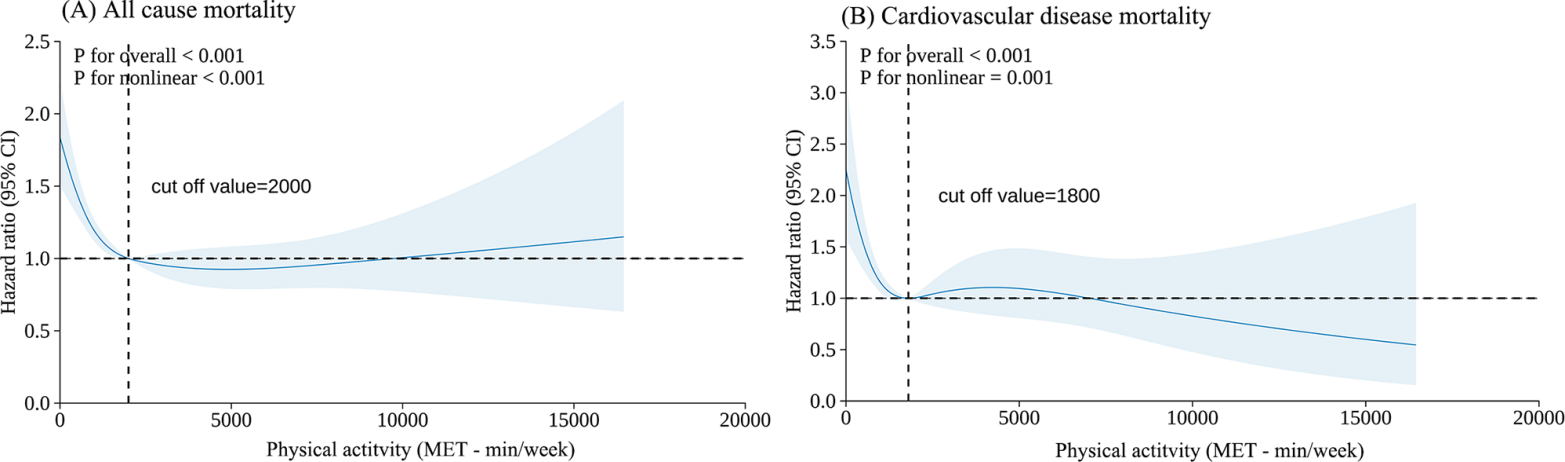
Dose–response associations between physical activity with all-cause (**a**), and cardiovascular disease mortality (**b**) restricted cubic splines were constructed with four knots located at the 5th, 35th, 65th, and 95th percentiles of each exposure. Adjusted hazard ratios (95% CI) were calculated with adjustment for age, sex, body mass index, mental health issue, employment, smoking status, drinking, sedentary behaviour, alone, hypertension, diabetes, and sleep

Compared to individuals who did not meet the recommended guidelines for MVPA, those who did achieve the recommended MVPA levels was exhibited a significantly lower risk of all-cause mortality after full adjustment (HR, 0.729; 95% CI, 0.640–0.831), Similarly, in the fulled adjusted model, adherence to the recommended MVPA was associated with a reduced risk of CVD mortality compared with those who did not achieve the recommended MVPA levels (HR, 0.786; 95% CI, 0.627–0.986) (Table 2). Across varying levels of PA, higher PA levels were associated with a reduced risk of both all-cause and CVD mortality when compared to the group with less than < 600 metabolic equivalent of task (MET)-min/week (Table S5). Similar associations between sleep score and PA with all-cause and CVD mortality were observed in stroke patients with first-reported events from 2006 to 2010 (Table S7). A significant nonlinear dose-response relationship (_Poverall_ < 0.001; _Pnonlinearity_ < 0.001) was identified between total PA and the decreased risk of all-cause and CVD mortality (Fig. 1). For stroke survivors, PA levels exhibit an L-shaped association with mortality, when PA levels were below 2000 MET-minutes per week, there was an association between higher PA and a reduced risk of all-cause and CVD mortality. However, above this threshold, PA levels were not significantly associated with all-cause and CVD mortality risks.

### Joint associations of physical activity and sleep score with mortality

**Fig. 2:**
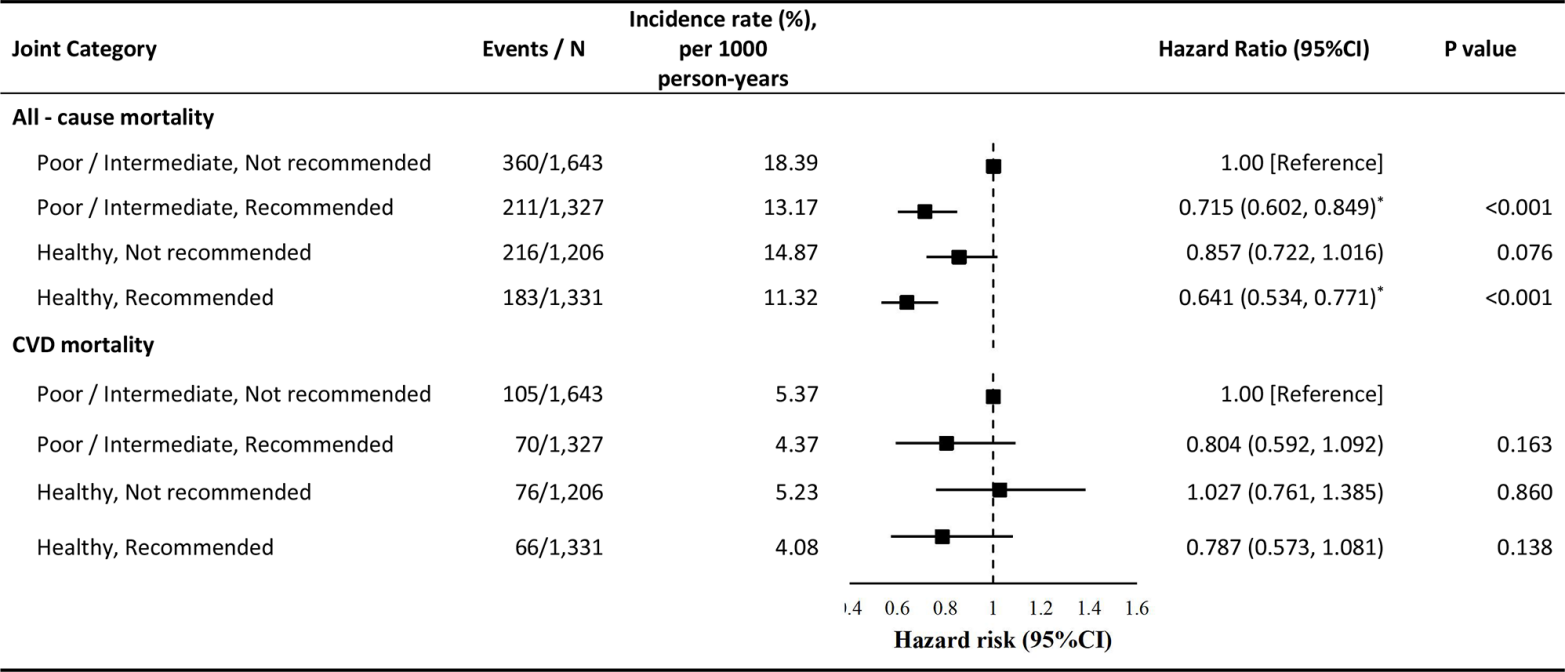
Joint associtions of sleep scope and moderate-to-vignorous physical activity with all-cause, cardiovascular disease mortality. Multivariable Cox model was adjusted for age, sex, body mass index, mental health issue, employment, smoking status, drinking, sedentary behavior, alone, hypertrnsion, and diabetes. *p<0.05

Figure 2 presents the HR for each combination of exposure relative to the reference group, which consists of individuals not meeting recommended MVPA guidelines and having poor/intermediate sleep score. After full adjustment, participants meeting MVPA recommendations but reporting poor/intermediate sleep score exhibited a significantly lower risk of all-cause mortality (HR, 0.715; 95% CI, 0.602–0.849). Addtionally, participants meeting MVPA recommendations and reporting healthy sleep scores also exhibited a significantly lower risk of all-cause mortality (HR, 0.641; 95% CI, 0.534–0.771). Sensitivity analysis revealed significant multiplicative interaction of sleep scores and MVPA on all-cause mortality (HR, 0.648; 95% CI, 0.500–0.841) (Table S6). However, this association was not observed for CVD mortality. Furthermore, the joint associations of PA and sleep scores with mortality were largely consistent among patients experiencing their first stroke from 2006 to 2010. (Table S8). We conducted association analyses in the competing risk model (Table S9), The outcomes aligned with the primary analysis, demonstrating that only physical activity showed a significant independent association with CVD mortality.

## Discussion

To the best of our knowledge, this is the first-ever prospective cohort study explore the joint effects of sleep comprehensive scores and PA on mortality outcomes among stroke survivors. Our study is also the most extensive investigation to date examining the joint effects of sleep scores and PA on all-cause and cardiovascular disease (CVD) mortality in this population. We present several key findings. Firstly, both PA levels and sleep scores were independently associated with all-cause mortality, witha PA threshold of 2,000 MET -mins /week potentially optimal for reducing all-cause mortality risk in stroke survivors. Secondly, there was a significant joint interaction between PA and sleep scores on all-cause mortality risk. Lastly, higher PA levels in stroke survivors seemed to attenuate the increase all-cause mortality risk associated with poor sleep.

Previous studies have primarily described the association between sleep quality and mortality risk based on a single sleep characteristic and have mainly focused on the general population, yielding conflicting findings [19–21]. Yin et al. concluded that insufficient sleep (< 7 h/day) was associated with an increased risk of all-cause mortality [20]. A prospective study showed that only long sleep duration (≥ 8 h/day) significantly increased the risk of all-cause mortality [19]. Due to the high prevalence of sleep disorders after stroke [22], in this study we use a novel comprehensive sleep score method [9] to explore the association of sleep and PA with mortality in stroke survivors. Similar to previous studies in the general population [4], we observed that among stroke survivors, healthy sleep scores were associated with a 12.7% decreased risk of all-cause mortality after adjusting for other coveriat. Our analysis of sleep patterns revealed a 32.5% and 19.2% increased risk of all-cause mortality in stroke survivors with long sleep duration (≥ 8 h/day) and frequent daytime dozing, respectively. Additionally, our study revealed healthy sleep scores were associated with a 2.5% decreased risk of CVD mortality, a finding that is consistent with the results of previous studies [12].

Similarly, our study identified a curvilinear relationship between PA levels and the mortality risk, aligning largely with previous findings in the general population [23]. Among stroke survivors meeting the recommended MVPA, we observed a 28.1% decreased risk of all-cause mortality after adjustment for other coveriat. Echoing the results of previous prospective studies [24], our data imply that achieving the recommended MVPA levels could be advantageous for stroke survivors. Additionally, our findings indicate that excessive PA (> 2,000 MET-mins/week) may not further reduce the risk of all-cause mortality, which is generally in agreement with previous findings [25].

While recent studies have suggested a synergistic impact of PA and sleep duration on health, the literature exploring the combined effects of sleep duration and PA on mortality risk is inconsistent [15]. One cohort study indicated that longer sleep duration negatively affected survival, particularly among individuals with low PA levels [26]. Conversely, another study showed that once PA levels reached 15 MET-hours per week, neither long nor shorte sleep duration were associated with the risk of all-cause and CVD mortality [15]. Furthermore, a recent study examining the association between PA and sleep score with mortality risk in the general population hinted at a potential synergy between these factors [4]. Our study echoes this, indicating that meeting guideline-recommended PA levels may mitigate some health consequences of poor/intermediate sleep score in stroke survivors [23], reducing the risk of all-cause mortality by 15.1–39.8%. However, no significant synergistic effect on the risk of CVD mortality was observed.

PA may mitigate the mortality risk associated with poor sleep through the following mechanisms. Poor/intermediate sleep is associated with obesity and inflammation [27–28], and sleep disorders are identified as risk factors for depression and dementia [29–30]. The adverse relationship between poor sleep and health is not fully understood, with one hypothesis suggesting that these associations might be non-causal and instead due to residual confounding factors such as fatigue, sleep fragmentation, substance use, or undiagnosed psychiatric disorders. Conversely, higher levels of PA are associated with reduced depression, cognitive decline, obesity, and CVD [31–33]. The beneficial health outcomes associated with PA may operate through enhanced metabolic adaptations, maintenance of stable circadian rhythms, promotion of healthy sleep patterns, and increased energy expenditure, thereby improving overall health outcomes [17].

This study is of significant clinical value and may guide clinical practice to include appropriate increases in PA levels for post-stroke survivors with poor sleep, in addition to conventional interventions such as medications. Furthermore, initiatives aimed at enhancing sleep quality could potentially decrease the prevalence of long-term negative outcomes for patients with constrained PA capacity due to post-stroke disabilities.

The strengths of the present study are the following: data are from a prospective cohort study with a large sample size, long-term follow-up, and extensive measurement of covariate, which allowed for a detailed analysis of PA and sleep scores. We used a novel sleep score method to include different sleep characteristics, providing a more appropriate method to study the combined effect of sleep and PA in stroke survivors. In addition, we focused on a specific population of stroke survivors to provide a rationale for this study. Our study has some potential limitations. First, our exposure metrics were measured by self-reporting, which is prone to reporting-and-recall bias and may underestimate the true nature of the association; however, previous studies have shown good agreement between sleep self-reporting and polysomnography [34]. Second, the present study is an observational study, and although adjusted for more confounding factors, we cannot exclude the possibility of unmeasured confounding variables. Particularly, stroke severity may influence both PA levels and sleep patterns through stroke-related complications, which could potentially confound the observed associations. Third, although the PA and sleep score categories showed statistically significant associations in the all-cause mortality analysis, the absence of significant associations and wide confidence intervals in the CVD mortality risk analysis may be attributable to the small number of positive events in these categories. The relatively small sample size in these combined groups may have affected the validity of our statistical tests, and therefore additional studies may be required to confirm our findings.

## Conclusion

In conclusion, our study revealed that poor sleep quality is associated with an elevated risk of all-cause mortality in stroke survivors. Importantly, stroke survivors meeting recommended MVPA levels exhibit lower mortality risk, even among those with poor sleep quality. These findings highlight the potential protective role of PA. Future intervention studies are needed to establish whether increasing PA to recommended levels can directly mitigate the mortality risk associated with poor sleep quality in this vulnerable population.

## Electronic supplementary material

Below is the link to the electronic supplementary material.

Supplementary Material 1

## Data Availability

The data that support the findings of this study are available from UK Biobank to any bona fide researchers who can apply to access the UK Biobank research resource. Researchers can apply via this link https://www.ukbiobank.ac.uk/enable-your-research/apply-for-access. However, requests can be forwarded to the corresponding authors regarding the particular datasets used and results produced in this study under the Material Transfer Agreement with UK Biobank.

## Abbreviations

CVD: Cardiovascular disease
BMI: Body mass index
HR: Hazard ratio
MET: Metabolic equivalent task
MVPA: Moderate-to-vigorous physical activity
NHS: National Health Service
PA: Physical activity
UKB: UK Biobank
WHO: World Health Organization
