# Supplementary material for: Joint effects of physical activity and sleep quality on all-cause and cardiovascular disease mortality in stroke survivors: a population-based cohort study from the UK-Biobank
Source: BMC Public Health. 2025 Apr 23;25:1502. doi: 10.1186/s12889-025-22588-5 (PMC12016377; doi:10.1186/s12889-025-22588-5)
Supplement: Supplementary file 1 — Supplementary Material 1 [file 12889_2025_22588_MOESM1_ESM.docx]

**Table S1** The Scoring System of Sleep.

| **Characteristics** | **UK BioBank Code** | **UK BioBank Questionnaire** | **Low risk Answer (%)** | **High risk Answer (%)** |
| --- | --- | --- | --- | --- |
| Chronotype | 1180 | Do you consider yourself to be? | Definitely a "morning" person;  More a "morning" than "evening" person. | More an "evening" than a "morning person;  Definitely an "evening" person. |
| Sleep Duration | 1160 | About how many hours sleep do you get in every 24 hours? (please include naps) | 7-8 hr/d. | <7 or >=9 hr/d. |
| Insomnia | 1200 | Do you have trouble falling asleep at night or do you wake up in the middle of the night? | Never/rarely; Sometimes. | Usually. |
| Snoring | 1210 | Does your partner or a close relative or friend complain about your snoring? | No. | Yes. |
| Daytime Sleepiness | 1220 | How likely are you to doze off or fall asleep during the daytime when you don't mean to?  (e.g. when working, reading or driving) | Never/rarely; Sometimes. | Often; All the Time. |

For each sleep factor, the participant received a score of 1 if he or she was classified as low risk for that factor or 0 if at high risk for that factor. Sleep scores were categorised into: poor, 0~1; intermediate, 2~3; healthy,4~5. For further information, please refer to the UK Biobank data showcase https://biobank.ndph.ox.ac.uk/showcase/search.cgi

**Table S2** The Resource and Definition of the Selected Covariates.

| Covariates | UK BioBank Code | Description | Category | Type |
| --- | --- | --- | --- | --- |
| Age (mean±SD) | 21022 | / | / | Continuous |
| Gender(n(%)) | 31 | / | female;  male | Categorical |
| BMI (n(%)) | 21001 | Trained nurses measured height (12144) and body weight (21002) during the baseline assessment. Body mass index (BMI) was derived from weight/height2 and then was classified using the WHO guidelines. | underweight (<18.5kg/m2); normal weight (18.5–24.9kg/m2); overweight (25.0–29.9kg/m2) ; obese (≥30.0kg/m2) | Categorical |
| Socioeconomic status (mean±SD) | 189 | The existing variable 'Townsend area deprivation index' (189) served as an indicator of socioeconomic status, with higher scores indicating greater socioeconomic deprivation. | / | Continuous |
| Sedentary behaviour (mean(SD)) | 1070, 1080,  1090 | The total daily hours of television viewing (1070), computer use (1080), and driving (1090) as a marker of sedentary behaviour. | / | Continuous |
| Mental health  issue (n(%)) | 2090, 2100 | Participants having ever seen a doctor (GP) (2090) or psychiatrist (2100) for nerves, anxiety, or depression were classified as having mental health issues. | Yes; No | Categorical |
| Employment (n(%)) | 6142, 3426, 826 | We derived four groups based on 'Current employment status' (6142), 'Job involves night shift work' (3426), and 'Job involves shift work' (826). | retired/not in the workforce;  employed not in shift work;  employed in night shift work;  employed in day shift work | Categorical |
| Cigarette  smoking (n(%)) | 20116 | The existing variable 'smoking status' (20116) was applied. | never;  previous smoker;  current smoker | Categorical |
| Alcohol  consumption (n(%)) | 20117, 1558 | The existing variable 'Alcohol drinker status' (20117) and ‘Alcohol intake frequency’(1558) was applied. | never;  previous drinker;  current drinker | Categorical |
| Alone(n(%)) | 709 | The existing variable 'Number in household' (709) was applied. | Yes; No | Categorical |
| Hypertension history(n(%)) | 131286 | The existing variable 'Date I10 first reported (essential (primary) hypertension)' (131286) was applied. | Yes; No | Categorical |
| Diabetes(n(%)) | 2443 | The existing variable 'Diabetes diagnosed by doctor' (2443) was applied. | Yes; No | Categorical |

**Table S3** Baseline characteristics of the study participants by categories of physical activity.

| **Characteristics** | **Physical activity** a | | | |
| --- | --- | --- | --- | --- |
|  | **All**  ***(N=5,507)*** | **Not recommended MVPA**  ***(N=2,849)*** | **Recommended MVPA**  ***(N=2,658)*** | ***p*** |
| age, mean (SD) | 60.12 (7.06) | 59.87 (7.00) | 60.39 (7.13) | 0.006 |
| Sex, n (%) |  |  |  | 0.792 |
| Male | 3,304 (60.00) | 1,704 (59.81) | 1,600 (60.20) |  |
| Female | 2,203 (40.00) | 1,145 (40.19) | 1,058 (39.80) |  |
| Body Mass Index (kg/m2), mean (SD) | 28.52 (5.10) | 29.03 (5.46) | 27.97 (4.64) | <0.001 |
| Body Mass Index (kg/m2), n (%) |  |  |  | <0.001 |
| Underweight (<18.5 kg/m2) | 102 (1.85) | 69 (2.42) | 33 (1.24) |  |
| Normal weight (18.5–24.9 kg/m2) | 1,260 (22.88) | 568 (19.94) | 692 (26.03) |  |
| Overweight (25.0–29.9 kg/m2) | 2,343 (42.55) | 1,191 (41.80) | 1,152 (43.34) |  |
| Obese (≥30.0 kg/m2) | 1,802 (32.72) | 1,021 (35.84) | 781 (29.38) |  |
| Socioeconomic status, mean (SD) | -0.74 (3.33) | -0.56 (3.44) | -0.93 (3.20) | <0.001 |
| Sedentary behaviour (hour/day), mean (SD) | 5.16 (2.72) | 5.42 (2.91) | 4.88 (2.48) | <0.001 |
| Mental health issue, n (%) |  |  |  | <0.001 |
| No | 3,284 (59.63) | 1,606 (56.37) | 1,678 (63.13) |  |
| Yes | 2,223 (40.37) | 1,243 (43.63) | 980 (36.87) |  |
| Employment status, n (%) |  |  |  | 0.001 |
| Retired/not in the workforce | 3,877 (70.40) | 2,008 (70.48) | 1,869 (70.32) |  |
| Employed not in shift work | 1,344 (24.41) | 725 (25.45) | 619 (23.29) |  |
| Employed in night shift work | 135 (2.45) | 56 (1.97) | 79 (2.97) |  |
| Employed in day shift work | 151 (2.74) | 60 (2.11) | 91 (3.42) |  |
| Cigarette smoking, n (%) |  |  |  | <0.001 |
| Never | 2,366 (42.96) | 1,171 (41.10) | 1,195 (44.96) |  |
| Previous smoker | 2,324 (42.20) | 1,198 (42.05) | 1,126 (42.36) |  |
| Current smoker | 817 (14.84) | 480 (16.85) | 337 (12.68) |  |
| Alcohol consumption, n (%) |  |  |  | 0.204 |
| Never | 312 (5.67) | 176 (6.18) | 136 (5.12) |  |
| Previous drinker | 381 (6.92) | 201 (7.06) | 180 (6.77) |  |
| Current drinker | 4,814 (87.42) | 2,472 (86.77) | 2,342 (88.11) |  |
| Living alone, n (%) |  |  |  | 0.352 |
| No | 4,193 (76.14) | 2,154 (75.61) | 2,039 (76.71) |  |
| Yes | 1,314 (23.86) | 695 (24.39) | 619 (23.29) |  |
| Hypertension history, n (%) |  |  |  | <0.001 |
| No | 2,917 (52.97) | 1,444 (50.68) | 1,473 (55.42) |  |
| Yes | 2,590 (47.03) | 1,405 (49.32) | 1,185 (44.58) |  |
| Diabetes history, n (%) |  |  |  | <0.001 |
| No | 4,821 (87.54) | 2,448 (85.92) | 2,373 (89.28) |  |
| Yes | 686 (12.46) | 401 (14.08) | 285 (10.72) |  |
| Sleep score, mean (SD) | 3.31 (1.09) | 3.23 (1.11) | 3.41 (1.06) | <0.001 |

Note: Continuous variables are presented as mean±SD; categorical variables are presented as n (%). a Recommended MVPA (≥150 min of MPA, or≥75 min of VPA, or equivalent combinations of both throughout the week). SD, standard deviation; n, total number; MVPA, moderate-to-vigorous physical activity; MPA, Moderate- intensity physical activity; VPA.Vigorous-intensity physical activity.

**Table S4** Associations of sleep pattern with all-cause and CVD mortality (n=5,507).

| Sleep pattern | All-cause mortality | | CVD mortality | |
| --- | --- | --- | --- | --- |
|  | **Model 1 HR (95% CI)^a^** | **Model 2 HR (95% CI)^b^** | **Model 1 HR (95% CI)^a^** | **Model 2 HR (95% CI)^b^** |
| Sleep duration |  |  |  |  |
| Short sleep duration | 1.129 (0.972, 1.312) | 1.105 (0.902, 1.222) | 1.076 (0.828, 1.398) | 1.001 (0.764, 1.297) |
| Normal sleep duration | 1.00 0[Reference] | 1.000 [Reference] | 1.00 0[Reference] | 1.000 [Reference] |
| Long sleep duration | 1.522 (1.286, 1.801)^*^ | 1.325 (1.118, 1.572)^*^ | 1.276 (0.937, 1.736) | 1.119 (0.819, 1.529) |
| Chronotype |  |  |  |  |
| Morning Chronotype | 1.000 [Reference] | 1.000 [Reference] | 1.000 [Reference] | 1.000 [Reference] |
| Evening Chronotype | 1.249 (1.091, 1.430)^*^ | 1.173 (1.024, 1.345)^*^ | 1.078 (0.849, 1.370) | 1.028 (0.808, 1.308) |
| Sleeplessness status |  |  |  |  |
| Rarely Sleeplessness | 1.000 [Reference] | 1.000 [Reference] | 1.000 [Reference] | 1.000 [Reference] |
| Sometimes Sleeplessness | 1.073 (0.908, 1.267) | 1.055 (0.893, 1.246) | 1.119 (0.829, 1.511) | 1.123 (0.831, 1.517) |
| Usually Sleeplessness | 1.138 (0.956, 1.354) | 1.001 (0.843, 1.201) | 1.345 (0.989, 1.829) | 1.209 (0.886, 1.652) |
| Snoring status |  |  |  |  |
| Usually Snoring | 1.000 [Reference] | 1.000 [Reference] | 1.000 [Reference] | 1.000 [Reference] |
| Rarely Snoring | 1.081 (0.951, 1.228) | 1.063 (0.935, 1.209) | 1.173 (0.936, 1.469) | 1.156 (0.922, 1.449) |
| Daytime dozing status |  |  |  |  |
| Rarely Daytime dozing | 1.000 [Reference] | 1.000 [Reference] | 1.000 [Reference] | 1.000 [Reference] |
| Sometimes Daytime dozing | 1.271 (1.108, 1.458)^*^ | 1.159 (1.002,1.332)^*^ | 1.232 (0.968, 1.569) | 1.131 (0.886,1.444) |
| Often Daytime dozing | 1.505 (1.183, 1.915)^*^ | 1.192 (1.005, 1.531)^*^ | 1.521 (1.001, 2.308)^*^ | 1.229 (0.801, 1.890) |

CI, confidence interval; CVD, cardiovascular disease; HR, hazard ratio.^*^: p < 0.05.

^a^ Adjusted for age and sex; ^b^ Further adjusted for body mass index, mental health issue, employment, smoking status, drinking, alone, sedentary behaviour, hypertension, diabetes, and physical activity.

**Table S5** Associations of physical activity with all-cause and CVD mortality (n=5,507).

|  | All-cause mortality | | CVD mortality | |
| --- | --- | --- | --- | --- |
|  | **Model 1 HR (95% CI)**^a^ | **Model 2 HR (95% CI)^b^** | **Model 1 HR (95% CI)**^a^ | **Model 2 HR (95% CI)^b^** |
| Physical activity |  |  |  |  |
| 0 < 600 MET-min/w | 1.000 [Reference] | 1.000 [Reference] | 1.000 [Reference] | 1.000 [Reference] |
| 600 < 1200 MET-min/w | 0.779 (0.650, 0.933)^*^ | 0.805 (0.671, 0.966)^*^ | 0.677 (0.491, 0.933)^*^ | 0.663 (0.477, 0.920)^*^ |
| ≥ 1200 MET-min/w | 0.564 (0.489, 0.651)^*^ | 0.631 (0.507, 0.786)^*^ | 0.527 (0.412, 0.673)^*^ | 0.453 (0.302, 0.681)^*^ |

CI, confidence interval; CVD, cardiovascular disease; HR, hazard ratio.^*^: *p* < 0.05.

^a^ Adjusted for age and sex; ^b^ Further adjusted for body mass index, mental health issue, employment, smoking status, drinking, alone, sedentary behaviour, hypertension, diabetes and sleep score.

**Table S6** Analyses on the interaction of sleep score ^a^ and physical activity ^b^ with all-cause, and cardiovascular disease mortality (n=5,507)

|  | **Poor/ Intermediate sleep score** | | **Healthy sleep score** | | **Hazard Ratio ^c^ (95% CI) for Sleep Scores within Strata of Physical Activity Level** |
| --- | --- | --- | --- | --- | --- |
|  | **Events/ N** | **HR (95% CI) ^*^** | **Events/ N** | **HR (95% CI) ^*^** |  |
| **All-cause mortality** |  |  |  |  |  |
| Not Recommended MVPA | 360/1,283 | 1.00 [Reference] | 216/990 | 0.867 (0.673, 1.115);  *P* = 0.266 | 0.862 (0.7323, 1.024);  *P* = 0.082 |
| Recommended MVPA | 211/1,116 | 0.715 (0.602, 0.849);  *P* < 0.001**^†^** | 183/1,148 | 0.648 (0.510, 0.841);  *P* < 0.001**^†^** | 0.894 (0.732,1.1093);  *P* = 0.248 |
| Hazard Ratio (95% CI) for Recommended MVPA within Strata of Sleep scores |  | 0.715 (0.602, 0.849);  *P* < 0.001**^†^** |  | 0.751 (0.608, 0.914);  *P* < 0.001**^†^** |  |
| **Cardiovascular disease mortality** |  |  |  |  |  |
| Not Recommended MVPA | 105/1,538 | 1.00 [Reference] | 76/1,130 | 1.132 (0.725, 1.767);  *P* = 0.586 | 1.042 (0.778, 1.433);  *P* = 0.812 |
| Recommended MVPA | 70/1,257 | 0.805 (0.593, 1.094);  P = 0.167 | 66/1,265 | 0.866 (0.549, 1.367);  *P* = 0.538 | 0.954 (0.681, 1.346)  *P* = 0.784 |
| Hazard Ratio (95% CI) for Recommended MVPA within Strata of Sleep scores |  | 0.811 (0.593, 1.147);  *P* = 0.171 |  | 0.773 (0.558, 1.071);  *P* = 0.119 |  |

Measure of interaction on additive scale: for all-cause death, RERI (95% CI) = 0.066 (-0.141, 0.273), AP (95% CI) =0.103 (-0.221, 0.425), and S = 0.841 (0.512, 1.379); for CVD death, RERI (95% CI) = -0.071 (-0.513, 0.372), AP (95% CI) = -0.081 (-0.591, 0.427), and S = 2.133 (0.001, 42.53). Measure of interaction on multiplicative scale: for all-cause death, HR (95% CI) = 0.648 (0.500, 0.841), P < 0.001; for CVD death, HR (95% CI) = 0.867 (0.549, 1.367), P = 0.359.

^a^ Sleep scores were categorized into: poor/ intermediate, 0~3; healthy, 4~5. ^b^ Physical activity levels were categorized based on MET-mins/wk from MVPA, regardless of total MET-mins/wk. ^*^Multivariable Cox model was adjusted for adjusted for age, sex, body mass index, mental health issue, employment, smoking status, drinking, sedentary behaviour, alone, hypertension, and diabetes. HR, hazard ratio; MVPA, moderate-to-vigorous physical activity. ^†^: *p* < 0.05.

**Table S7** The independent (and mutually adjusted) associations of physical activity and sleep scores with mortality in the new stroke population, 2006-2010 (n=1,997).

|  | **Number of cases** | **Number of total** | **Person-years** | **Incidence density (1000 person-year)** | **Model 1** | **Model 2** |
| --- | --- | --- | --- | --- | --- | --- |
| **All-cause Mortality** | | | | | | |
| Sleep Scores (categorical) | | | | | | |
| Poor/Intermediate | 209 | 1,083 | 12,872.35 | 16.23 | Ref. | Ref. |
| Healthy | 130 | 914 | 11,026.30 | 11.78 | 0.730 (0.586, 0.909)**^*^** | 0.793 (0.634, 0.991)**^*^** |
| Sleep Scores (continuous) |  |  |  |  | 0.864 (0.782, 0.954)**^*^** | 0.918 (0.829, 1.000)**^*^** |
| Physical Activity (categorical) | | | | | | |
| Not recommended MVPA | 195 | 988 | 11,756.32 | 16.59 | Ref. | Ref. |
| Recommended MVPA | 144 | 1,009 | 12,142.33 | 11.85 | 0.666 (0.537, 0.826)**^*^** | 0.674 (0.541, 0.839)**^*^** |
| Physical Activity (continuous) |  |  |  |  | 0.999 (0.999, 1.000) | 0.999 (0.999, 1.000)**^*^** |
| **CVD Mortality** | | | | | | |
| Sleep Scores (categorical) | | | | | | |
| Poor/Intermediate | 77 | 1,083 | 12,872.35 | 5.98 | Ref. | Ref. |
| Healthy | 39 | 914 | 11,026.30 | 3.53 | 0.592 (0.403, 0.872)**^*^** | 0.628 (0.424, 0.930)**^*^** |
| Sleep Scores (continuous) |  |  |  |  | 0.822 (0.694, 0.974)**^*^** | 0.860 (0.723, 1.024)**^*^** |
| Physical Activity (categorical) | | | | | | |
| Not recommended MVPA | 62 | 988 | 11,756.32 | 5.27 | Ref. | Ref. |
| Recommended MVPA | 54 | 1,009 | 12,142.33 | 4.44 | 0.782 (0.543, 1.127) | 0.807 (0.556, 1.171) |
| Physical Activity (continuous) |  |  |  |  | 0.998 (0.997, 1.000) | 0.999 (0.999, 1.000) |

^a^ Physical activity were categorization based on public health guidelines: Recommended MVPA (≥150 min of MPA, or≥75 min of VPA, or equivalent combinations of both throughout the week); Not recommended MVPA. Sleep scores were categorized into: poor/intermediate, 0~3; healthy, 4~5.
Model1: Adjusted for age and sex; Model2: Further adjusted for BMI, mental health issue, employment, smoking status, drinking, sedentary behaviour, alone, hypertension, diabetes and mutually adjusted for sleep scores or physical activity levels as appropriate. ^*^: *p* < 0.05.
CVD, cardiovascular disease; MVPA, moderate-to-vigorous physical activity; MPA, Moderate- intensity physical activity; VPA.Vigorous-intensity physical activity.

**Table S8** Joint associations of sleep score and moderate-to-vigorous physical activity with all-cause, cardiovascular disease mortality in the new stroke population, 2006-2010 (N=1,997).

| **Joint Category** | **Events / N** | **Person - years** | **Incidence rate (%), per 1000 person-years** | **Model 1**  **Hazard Ratio (95%CI)** | **Model 2**  **Hazard Ratio (95%CI)** |
| --- | --- | --- | --- | --- | --- |
| **All - cause mortality** |  |  |  |  |  |
| Poor/ intermediate, Not recommended | 124 / 574 | 6,800.42 | 18.23 | Ref. | Ref. |
| Poor/intermediate, Recommended | 85 / 509 | 6,017.93 | 14.12 | 0.712 (0.540, 0.938)^*^ | 0.7687 (0.520, 0.909)^*^ |
| Healthy, Not recommended | 71 / 414 | 4,955.90 | 14.23 | 0.791 (0.591, 1.059) | 0.811 (0.603, 1.089) |
| Healthy, Recommended | 59 / 500 | 6,070.39 | 9.71 | 0.502 (0.368, 0.685)^*^ | 0.530 (0.385, 0.728)^*^ |
| **CVD mortality** |  |  |  |  |  |
| Poor/intermediate, Not recommended | 41 / 574 | 6,800.42 | 6.02 | Ref. | Ref. |
| Poor/ intermediate, Recommended | 36 / 509 | 6,017.93 | 5.98 | 0.903 (0.577, 1.415) | 0.7884 (0.561, 0.393) |
| Healthy, Not recommended | 21 / 414 | 4,955.90 | 4.23 | 0.701 (0.414, 1.186) | 0.711 (0.417, 1.208) |
| Healthy, Recommended | 18 /500 | 6,070.39 | 2.96 | 0.460 (0.264, 0.801)^*^ | 0.484 (0.274, 0.855)^*^ |

Model1: Adjusted for age and sex; Model2: Further adjusted for BMI, mental health issue, employment, smoking status, drinking, sedentary behaviour, alone, hypertension, diabetes and mutually adjusted for sleep scores or physical activity levels as appropriate. *: p < 0.05. CVD, cardiovascular disease.

**Table S9** The association between the two primary exposures (MVPA and sleep scores) and CVD mortality in a competing risk model.

|  | **SHR (95%CI)** | |
| --- | --- | --- |
|  | **Model 1** | **Model 2** |
| **Sleep Scores (categorical)** |  |  |
| Poor/Intermediate | Ref. | Ref. |
| Healthy | 0.954 (0.765, 1.176) | 1.011 (0.812, 1.269) |
| **Sleep Scores (continuous)** | 0.948 (0.861, 1.064) | 0.991 (0.894, 1.102) |
| **Physical Activity (categorical)** |  |  |
| Not recommended MVPA | Ref. | Ref. |
| Recommended MVPA | 0.814 (0.651, 1.000)^*^ | 0.808 (0.648, 1.008) |
| **Physical Activity (continuous)** | 0.999 (0.998, 1.000)^*^ | 0.999 (0.999, 1.000)^*^ |
| **Joint Category** |  |  |
| Poor/intermediate, Not recommended | Ref. | Ref. |
| Poor/ intermediate, Recommended | 0.835 (0.612, 1.124) | 0.831 (0.614, 1.127) |
| Healthy, Not recommended | 0.991 (0.734, 1.321) | 1.042 (0.771, 1.409) |
| Healthy, Recommended | 0.781 (0.571, 1.057) | 0.815 (0.591, 1.124) |

^a^ Physical activity were categorization based on public health guidelines: Recommended MVPA (≥150 min of MPA, or≥75 min of VPA, or equivalent combinations of both throughout the week); Not recommended MVPA. Sleep scores were categorized into: poor/intermediate, 0~3; healthy, 4~5. MVPA: moderate-to-vigorous physical activity; SHR: subdistribution hazard ratios; CI: confidence interval.


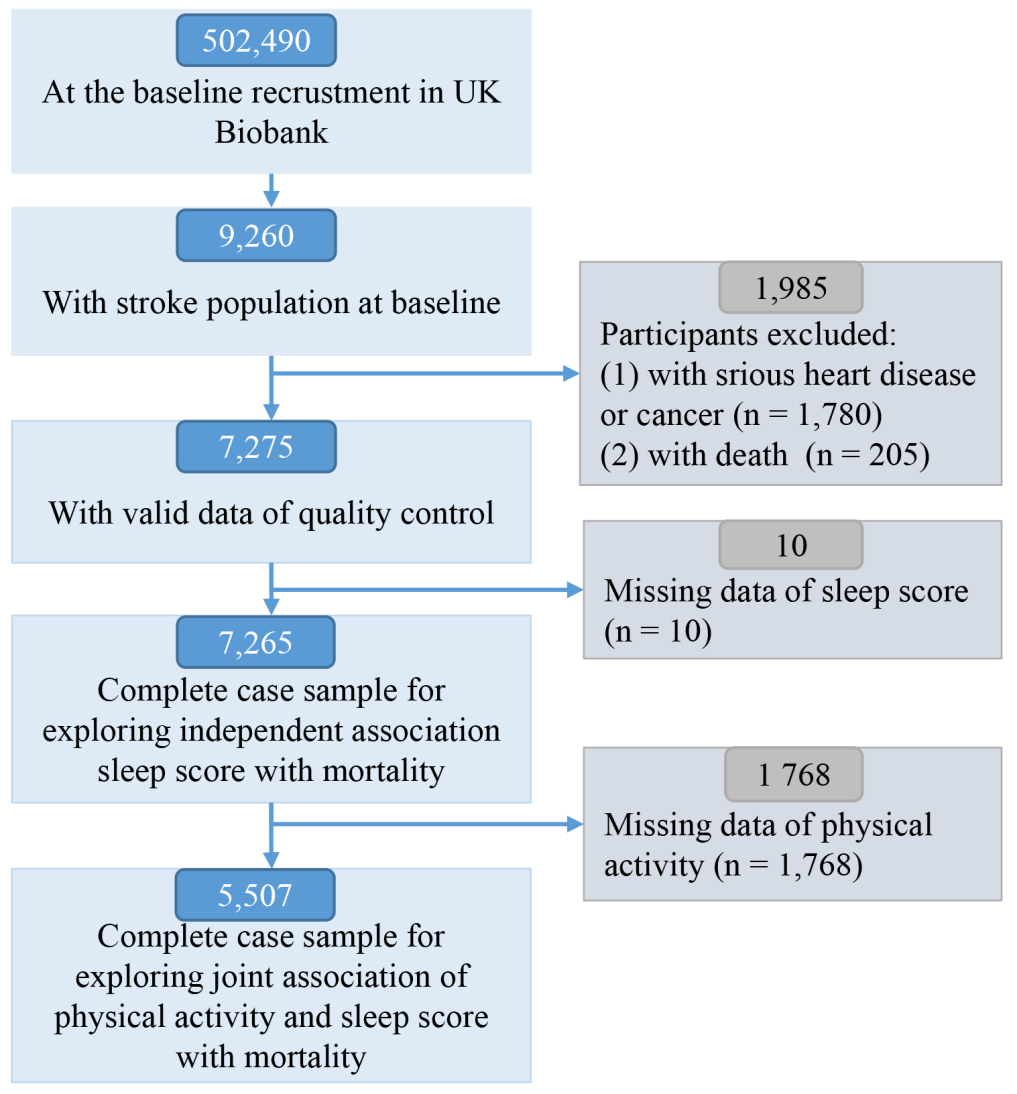


**Figure S1** Flowchart of participant enrolment.


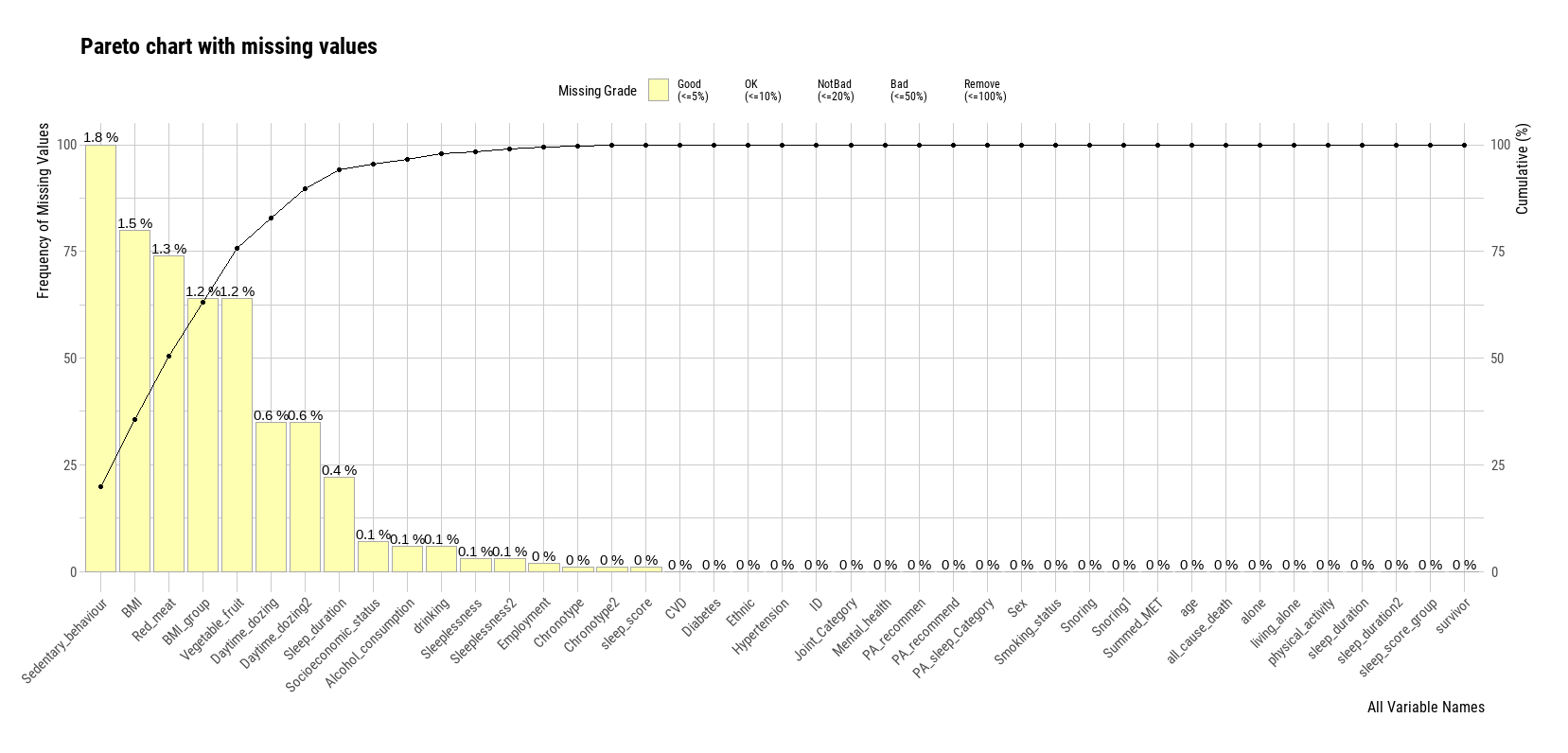


**Figure S2** Pareto chart with missing values.


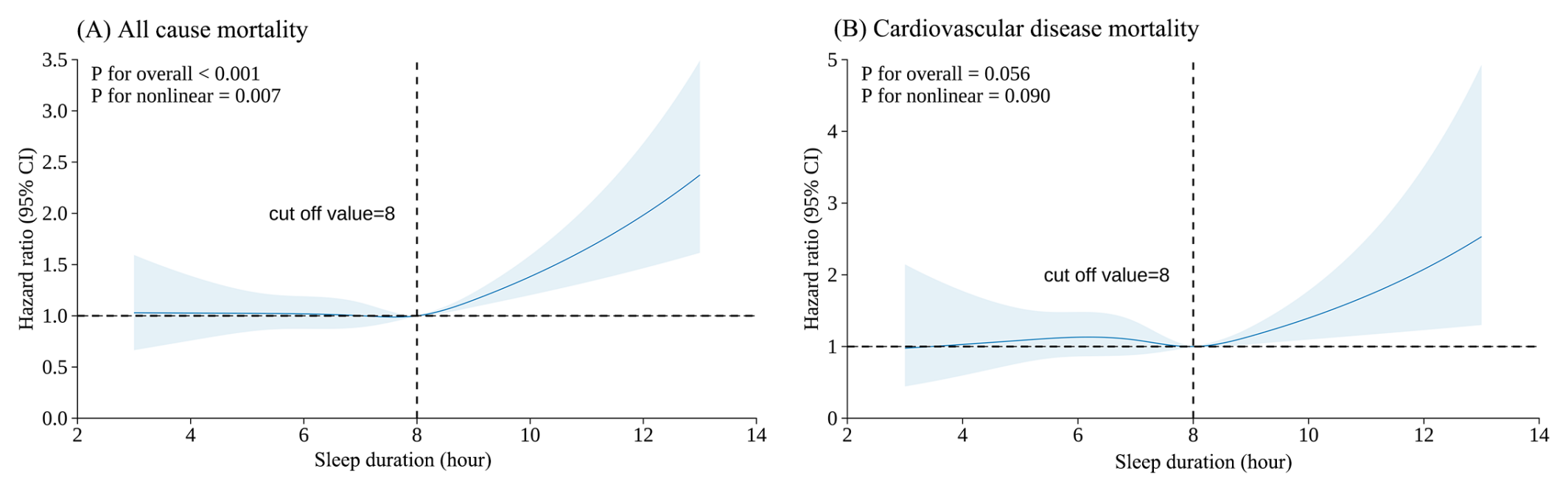


**Figure S3** Dose–response associations between sleep duration with all-cause (a), and cardiovascular disease mortality (b). Restricted cubic splines were constructed with four knots located at the 5th, 35th, 65th, and 95th percentiles of each exposure. Adjusted hazard ratios (95% CI) were calculated with adjustment for age, sex, body mass index, mental health issue, employment, smoking status, drinking, sedentary behaviour, alone, hypertension, diabetes, and physical activity.
